# Psychiatric patients at the emergency department: factors associated with length of stay and likelihood of hospitalization

**DOI:** 10.1007/s11739-021-02820-x

**Published:** 2021-08-11

**Authors:** Enrica Marzola, Elisa Duranti, Carlotta De-Bacco, Enrico Lupia, Vincenzo Villari, Giovanni Abbate-Daga

**Affiliations:** 1 Eating Disorders Center, Department of Neuroscience “Rita Levi Montalcini”, University of Turin, Via Cherasco 11, 10126 Turin, Italy; 2 Division of Emergency Medicine and High Dependency Unit, AOU Città della Salute e della Scienza di Torino, Molinette Hospital, Turin, Italy; 3 Department of Medical Sciences, University of Turin, Turin, Italy; 4 Neuroscience and Mental Health Department, AOU Città della Salute e della Scienza, Torino, Italy

**Keywords:** Anxiety, Depression, Suicidal ideation, Consultation, Intoxication

## Abstract

Emergency department (ED) care for psychiatric patients is currently understudied despite being highly utilized. Therefore, we aimed to analyze psychiatric patients' length of stay (LOS) and LOS-related factors at the ED and to investigate and quantify the likelihood of being hospitalized after an emergency psychiatric evaluation. Charts of 408 individuals who sought help at the ED were retrospectively assessed to identify patients' sociodemographic and clinical data upon ED admission and discharge. All interventions performed at the ED (e.g., medications, hospitalization, clinical advice at discharge) were collected as well. The LOS for psychiatric patients was relatively short (6.5 h), and substance/alcohol intoxication was the main factor impacting LOS. Upon ED arrival, hospitalized patients were mostly men, most often had a yellow/severe triage code, and most often had a positive history of psychiatric illness, psychotic symptoms, euphoric mood, or suicidal ideation. Manic symptoms and suicidal ideation were the conditions most frequently leading to hospitalization. Given the paucity of real-world data on psychiatric patients’ LOS and outcomes in the ED context, our findings show that psychiatric patients are evaluated in a reasonable amount of time. Their hospitalization is mostly influenced by clinical conditions rather than predisposing (e.g., age) or system-related factors (e.g., mode of arrival).

## Introduction

Poor mental health puts a severe burden on sufferers and health systems alike, and the emergency department (ED) often becomes a crucial component of psychiatric patients’ clinical pathways. According to recent data from the Ministry of Health of Italy, 837,027 psychiatric patients are seen by the Italian National Health System, with an average standardized ratio of 166.6/10,000 [1]. In 2018, the overall number of evaluations at EDs because of psychiatric reasons was 617,326 patients (in 2016, 592,226), representing 3% of all ED admissions registered in our country (*n* = 20,853,449). However, available data on the proportion of psychiatric patients out of all those who seek help at the ED vary across countries, with studies reporting proportions ranging from 4% [2] to 12.5% [3], ultimately reflecting much different health care systems. Additionally, it has been reported that psychiatric consultations at the ED show some peculiarities, including duration, with data from the National Center for Health Statistics showing that the average duration of ED mental health visits was 42% greater than that of non-mental health visits [4].

Relatedly, for medical patients, the length of stay (LOS) at the ED is receiving growing attention because it is related to negative outcomes, including increased mortality and crowding [5], greater costs [6], and decreased patient satisfaction [7]. Therefore, LOS has become a tool to measure overall emergency care quality [8]. However, the literature has mainly focused on general medical patients, while the LOS of psychiatric patients in the ED has received less attention [9, 10]. In addition, the main data on this topic in the scientific literature are contrasting, with some studies showing that psychiatric patients tend to stay longer at the ED than medical patients [11, 12] and others reporting the opposite result [13]. A related line of research is on factors potentially influencing LOS, but only a few studies are available on psychiatric patients, which have yielded mixed results [14, 15]. It should be noted that data from Europe are lacking (for example, in Germany, emergency medicine is not a specialty), and psychiatric patients are sometimes not admitted through the ED [16], so data are elusive.

Another important issue for psychiatrists who work in the ED is the timely decision to admit or discharge patients seeking help for a psychiatric emergency. Again, concerning hospitalization following ED admission, data in the literature vary, possibly mirroring differences across mental health systems. The severity of illness and being diagnosed with psychosis or bipolar disorder were found to significantly predict hospitalization [17, 18]. The literature also consistently proposes symptoms including active suicidality, consciousness alterations, hallucinations, delusions, psychomotor agitation and inhibition, pantoclastic crises, and danger to self or others, as robustly associated with hospitalization [19]. In contrast, anxiety is reported to be negatively associated with hospitalization [20], while the more debated factors include age [17, 21, 22], previous hospitalizations [17, 20], sex, social support (including being married), and socioeconomic status [18]. Therefore, to help clinicians make difficult clinical decisions, it would be helpful to better investigate those baseline features that are associated with a greater likelihood of being hospitalized.

Therefore, in an attempt to fill the aforementioned gaps in the literature, we had the primary aim of analyzing the LOS (by cumulative survival analysis of time to discharge and factors influencing LOS) at one of the largest EDs in Italy, focusing on patients’ trajectory for each main psychiatric clinical presentation. As a secondary outcome, we aimed to investigate and quantify the likelihood of being hospitalized after a psychiatric evaluation at the ED, according to the clinical baseline presentation.

## Materials and methods

### Participants

Charts of individuals who sought help at the Emergency Department at the Città della Salute e della Scienza Hospital, University of Turin, Italy, from April to September 2018 were retrospectively assessed by two researchers (E.D. and E.M.), with good interrater reliability (92%). The last author (G.A.D.) was contacted in case of disagreement. Clinical charts at the ED typically included the following information: patients' age, sex, sociodemographic data, triage code for urgent care, reason for ED admission, already known diagnosis, symptoms that required ED admission, main data on the patient’s medical/psychiatric history, past and current medications, allergies, and previous hospitalizations. All interventions performed at the ED (e.g., medications, hospitalization, clinical advice at discharge) were specified as well. However, according to the level of emergency, not all anamnestic data could be filled out by the physicians. Inclusion criteria were (a) a psychiatric consultation being asked for by the patients themselves (e.g., the patients’ primary reason for ED admission), a law enforcement agency, or the ED physician; and (b) age > 18 years. Overall, 408 patients were included in the study during a total of 130 12-h shifts conducted by all psychiatrists working at the Academic Psychiatry Ward of the Città della Salute e della Scienza urban academic hospital in Turin, Italy. Out of a total of 130 shifts considered, 72 were day shifts, while 58 were night shifts.

Given the retrospective design of this study, no written informed consent was required by the ethics committee of our institution. Our study was approved with number CS2/843 by the Ethics Committee of the Città della Salute e della Scienza Hospital, Turin, Italy.

### Methods

According to the Italian National Health System, there are no barriers to patient access to the ED, independent of their insurance, economic, cultural, or language status. According to the organization of the Città della Salute e della Scienza hospital of the University of Turin, once patients are admitted to the hospital triage, they are referred, as a frontline intervention, to intensive care (e.g., suicidal attempts) or to either emergency medicine or emergency surgery. The triage code is assigned by a trained nurse on a priority basis, indicating how rapidly an incoming patient needs to be clinically evaluated. Afterward, all kinds of consultations can be required, including psychiatric consultations. Moreover, patients can arrive at the triage area specifically asking for a psychiatric visit or can be referred to the psychiatric consultation by the frontline physicians/surgeons.

In the Italian National Health System, nearly all hospitals are public and receive funds from taxes. Every individual has a general practitioner and is covered by the National Health System; therefore, insurance is not needed for access to the healthcare system, in either standard (e.g., GP) or emergency conditions (e.g., need for ambulance and ED). The majority of those who are seeking help at the ED are in an emergency condition; some patients can be referred to the ED by their GP when needing a diagnostic/therapeutic procedure that cannot be postponed. Ambulances are staffed with paramedics, and in some cases, a physician is on board as well; an ambulance for an emergency is free of charge for those who need it, independent of their economic/insurance status. The Città della Salute e della Scienza hospital is one of the largest hospitals in Italy and has an estimated catchment population of approximately 250,000 patients. Given its key role, approximately 75% of the patients in this study were not from the local catchment area.

Length of stay (LOS) was defined as the difference between the patient’s admission (e.g., patient's entrance to the unit) and disposition times (e.g., the decision to hospitalize or discharge the patient after performing clinical and diagnostic evaluations and urgent treatments).

### Statistical analysis

The SPSS 26.0 statistical software package (IBM SPSS Statistics for Windows, Version 26.0. Armonk, NY: IBM Corp) was used for data analysis. Student's *t* test for independent samples was used to evaluate the differences in continuous variables between groups (e.g., hospitalized versus discharged patients). The Mann–Whitney test was run to compare groups (e.g., very long LOS versus shorter LOS) when normality assumptions were not met. Cumulative survival analysis was done to measure the time to discharge, and Kaplan–Meier analysis with the log rank (Mantel–Cox) test was run to investigate the time-to-event relationship with either hospitalization or discharge as the outcomes for each psychiatric symptom reported upon ED admission. Binary logistic regression analyses were conducted to ascertain the association between baseline conditions and hospital admission after ED psychiatric consultation.

## Results

Based on our thorough investigation of the clinical charts, the results were divided into the following categories: (a) sociodemographic and clinical characteristics of the sample; (b) length of stay at the ED and its predictors; (c) hospitalization as an outcome of the ED access; and (d) discharge as an outcome of the ED access.

### Sociodemographic and clinical characteristics of the sample

The participants’ mean age was 46.31 ± 17.12 years [range: 18–88 years]. The participants were mostly Italian (89.9%), women (55.4%), and living in Turin (66.8%). The majority of patients sought treatment with a moderate severity of symptoms, namely, a green triage code (66.9%); most lived mostly outside the hospital’s catchment area (75.3%); family members were the most represented career category upon presentation at the ED (53%). Overall, 94.9% of patients received only one psychiatric evaluation, and a small minority of patients left the ED without waiting for the psychiatric assessment (2.2%). See Table 1 for all details.

**Table 1:** Variables concerning the access to the Emergency Department for patients who received a psychiatric evaluation at the ED

	Total sample *n* = 408
	*n* (%)
Level of emergency severity/triage codes	
Mild/White	7 (1.7)
Moderate/Green	273 (66.9)
Severe/Yellow	120 (29.4)
Very severe/Red	8 (2)
Local catchment area	110 (27)
Mode of ED arrival	229 (56.1)
Family members	121 (52.8)
Ambulance personnel	83 (36.2)
Law enforcement agency	11 (4.8)
Others	14 (6.2)
Number of psychiatric assessments during the ED stay	
< 2	387 (94.9)
≥ 2	21 (5.1)
Patients leaving the ED without waiting for the psychiatric evaluation	10 (2.5)

The vast majority of patients were referred to psychiatric consultations by emergency medicine physicians (*n* = 380, 93.1%) and less frequently by intensive care (*n* = 14; 3.4%) and surgery (*n* = 14; 3.4%) frontline clinicians. Two hundred forty-seven patients (60.5%) underwent blood tests, 210 (51.5%) underwent EKG, 42 (10.3%) underwent head CT, 26 (6.2%) underwent chest X-ray, and 6 (1.5%) underwent abdominal ultrasound examination.

The main findings from the participants’ clinical evaluation upon ED arrival are shown in Table 2. The majority (*n* = 312; 76.5%) of patients included in this study sought specific psychiatric help at the ED, while the remaining participants (*n* = 96, 23.5%) required ED admission because of an organic reason but then, according to the frontline physician’s/surgeon’s decision, required a psychiatric assessment. The most frequent psychiatric symptoms were anxiety (*n* = 118, 37.8%), followed by psychomotor agitation (*n* = 54, 17.3%) and depression (*n* = 32, 10.3%). A high proportion of participants showed a positive history of psychiatric illness (*n* = 303; 74.3%) and were already in the care of public psychiatric service (*n* = 220; 53.9%) or were already using psychiatric medications (*n* = 249; 61%).

**Table 2:** Patients' main reasons for seeking the Emergency Department intervention as assessed by the hospital triage nurses

	Total sample *n* = 408
	*n* (%)
Psychiatric main symptoms at triage	312 (76.5)
Anxiety	118 (37.8)
Psychomotor agitation	54 (17.3)
Depression	32 (10.3)
Patients' non-specific request of psychiatric consultation and/or psychiatric medications	29 (9)
Substance/alcohol intoxication	17 (5.4)
Psychosis	15 (4.9)
GP's request for hospitalization	15 (4.9)
Insomnia	13 (4.3)
Suicidal self-injury ideation	8 (2.6)
Suicidal attempt	8 (2.6)
Substance abuse	3 (0.9)
Organic main symptoms at triage then followed by psychiatric consultation	96 (23.5)
Medical	84 (87.4)
Surgical	12 (12.6)
Positive psychiatric history	303 (74.3)
In charge of a psychiatric service	220 (53.9)
National Health System	128 (58.1)
Private practice	55 (25)
Academic psychiatric services	19 (8.7)
Combined	18 (8.2)
Previous admission to the same ED	161 (39.4)
< 1 month	55 (34.2)
> 1 month < 6 months	56 (34.8)
> 6 months < 1 year	22 (13.7)
> 1 year	28 (17.3)

### Length of stay at the ED and its predictors

#### Length of stay at the ED

**Fig. 1:**
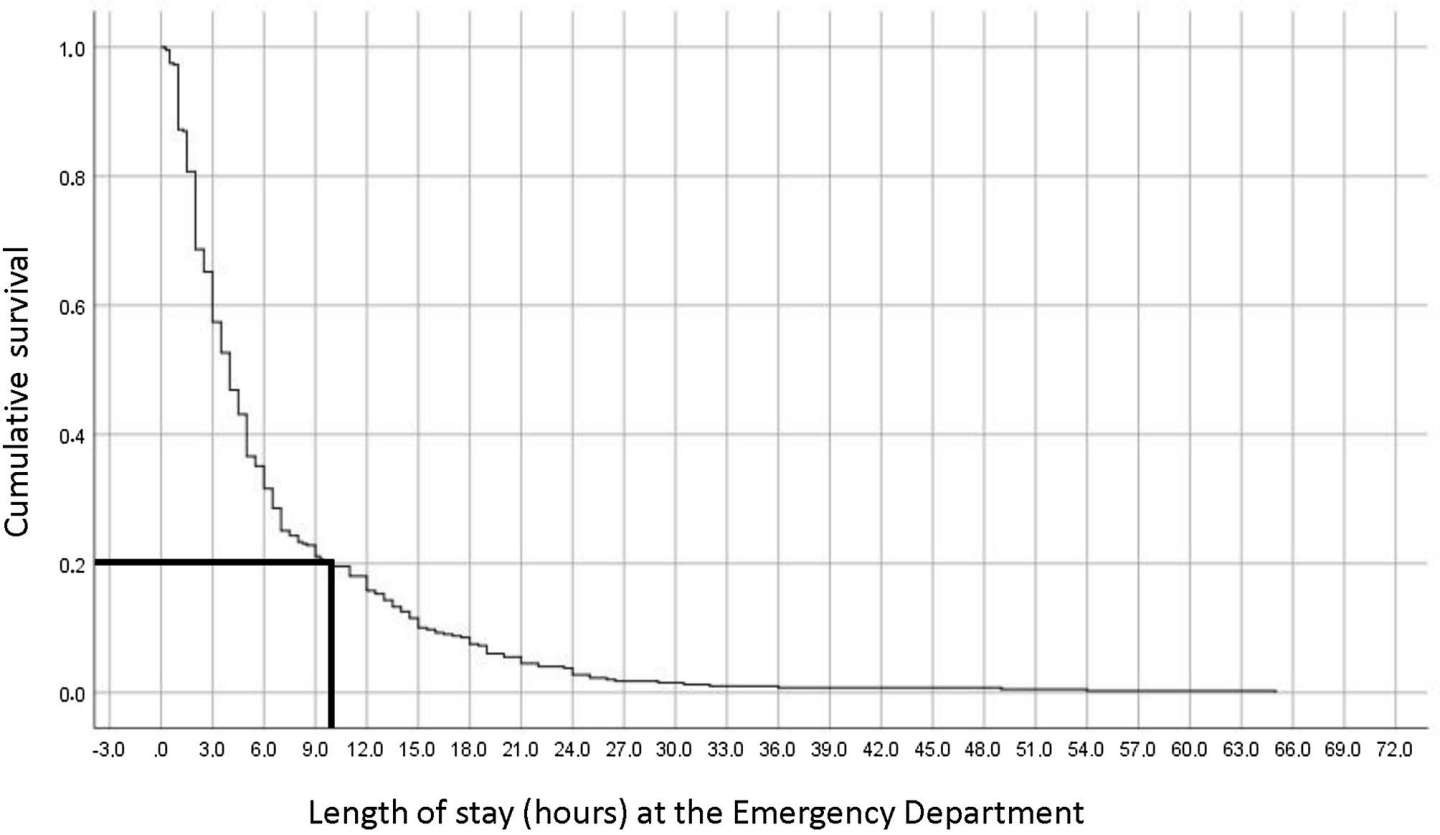
Time-to-discharge curve for psychiatric patients admitted to the ED

Participants stayed at the ED for a mean of 6.5 ± 7.6 h, with a wide range of times (0.3–65 h). As shown in Fig. 1, 2.5 h were needed to make a decision (e.g., hospitalization versus discharge) for 20% of the ED patients, and after 9 h in the ED, 80% of the admissions were resolved.

As shown in Table 3, patients with a long LOS (> 6.5 h) differed from those with a short LOS (≤ 6.5 h) only in the rates of anxiety and substance/alcohol intoxication (see Table 3). Similarly, patients with a very long LOS (i.e., LOS > 24 h) did not differ from those with a shorter LOS concerning any variables but the rates of red triage codes, anxiety symptoms, and substance/alcohol intoxication (see Table 3 for all details).

**Table 3:** Clinical differences between patients with different length of stay (LOS) at the ED

	Total sample (*n* = 408)					
	LOS ≤ 6.5 h *n* = 290	LOS > 6.5 h *n* = 118	Statistics	LOS < 24 h *n* = 395	LOS > 24 h *n* = 13	Statistics
	*n* (%)	*n* (%)	*p*	*n* (%)	*n* (%)	*p*
Gender			0.913			0.761
Females	160 (55.2)	66 (55.9)		218 (55.3)	8 (63.6)	
Males	130 (44.8)	52 (44.1)		177 (44.7)	5 (36.4)	
Nationality			0.295			0.315
Italian	264 (91.3)	102 (86.4)		353 (89.9)	10 (81.8)	
Other	26 (8.7)	16 (13.6)		42 (10.1)	3 (18.2)	
Age, years^a^	46 (16.4)^a^	46.6 (19.6)^a^	0.764^a^	45.9 (17.2)^a^	54.9 (21.4)^a^	0.065^a^
Local catchment area	79 (27.2)	31 (26.3)	0.902	107 (27.1)	1 (7.6)	0.301
Level of emergency severity/triage codes			0.282			**0.047**
Mild/White	4 (1.4)	3 (2.5)		6 (1.3)^b^	0 (0)^b^	
Moderate/Green	200 (69)	73 (61.9)		262 (67.1)^b^	7 (54.5)^b^	
Severe/Yellow	82 (28.3)	38 (32.2)		120 (30.1)^b^	4 (27.3)^b^	
Very severe/Red	4 (1.3)	4 (3.4)		7 (1.5)^b^	2 (18.2)^c^	
Anxiety	210 (72.4)	71 (60.2)	**0.018**	273 (69.1)	3 (23)	**0.005**
Substance/alcohol intoxication	13 (4.5)	23 (19.5)	** < 0.001**	31 (7.5)	5 (38)	**0.001**
Psychomotor agitation	32 (11)	10 (8.5)	0.479	40 (10.1)	2 (15.4)	0.234
Mood alterations	170 (58.6)	66 (55.9)	0.659	227 (57.4)	6 (46.1)	1
Hallucinations	9 (3.1)	6 (5.1)	0.385	13 (3.3)	2 (15.4)	0.059
Delusions	41 (14.1)	12 (10.2)	0.331	52 (13.1)	1 (7.6)	1
Suicidal ideation	26 (9)	7 (5.9)	0.329	32 (8.2)	1 (7.6)	1

^a^Mean (SD) and *t* test statistics applied

^b^Comparison of column proportions

^c^Comparison of column proportions

#### Differences in length of stay at the ED according to clinical presentation upon admission

As shown in Table 4, those with anxiety were admitted to the ED and discharged significantly sooner than those without anxiety. Those with suicidal ideation were admitted earlier but discharged later than those without suicidal ideation. In the case of psychomotor agitation and delusions, patients were admitted significantly sooner than those not reporting such clinical presentations (see Table 4).

**Table 4:** Differences in length of stay at the ED according to clinical presentation upon admission

	Total sample (*n* = 408)			
	Time-to-discharge (hours)	Log rank test *p*	Time-to-admission (hours)	Log rank test *p*
	Mean (SD)		Mean (SD)	
Anxiety		** < 0.001**		**0.026**
Yes	7.5 (0.5)		18 (1.4)	
No	18.2 (2.7)		19.7 (2.9)	
Suicidal ideation		** < 0.001**		** < 0.001**
Yes	19.2 (2.6)		7.3 (1.5)	
No	9.6 (0.8)		26 (3.1)	
Substance/alcohol intoxication		** < 0.001**		0.446
Yes	24.6 (4.2)		27.5 (5.6)	
No	8.8 (0.8)		19.7 (2.2)	
Psychomotor agitation		0.12		** < 0.001**
Yes	13.1 (2.2)		10.6 (2.1)	
No	10.3 (0.9)		23 (2.7)	
Delusions		0.21		** < 0.001**
Yes	10.8 (1.8)		10.6 (1.9)	
No	10.3 (0.9)		23.9 (2.8)	
Hallucinations		0.11		0.3
Yes	15.9 (4.1)		13.2 (3.1)	
No	10.1 (0.9)		23.3 (2.7)	
Mood alterations		0.77		0.51
Yes	10.7 (1.3)		22.7 (3.2)	
No	10.1 (1.3)		20.1 (3.2)	

#### Predictors of LOS at the ED

Factors associated with a long LOS (> 6.5 h) for patients requiring a psychiatric evaluation at the ED were investigated. No patient characteristics upon arrival, including age (*p* = 0.764), nationality (*p* = 0.331), mode of arrival (*p* = 0.111), positive psychiatric history (*p* = 0.16), and triage code (*p* = 0.344), were associated with a long LOS.

Concerning clinical presentations upon arrival, since patients with long (> 6.5 h) and short (≤ 6.5 h) LOSs differed in anxiety and substance/use intoxication, we controlled the subsequent analyses for these conditions. As a result, after these adjustments, only alcohol/drug intoxication was demonstrated to favor a long LOS (Wald's test = 19.02, OR = 5.02 [95% CI 2.4–10.4], *p* < 0.001).

### Hospitalization as an outcome of the ED access

#### Baseline differences between hospitalized and discharged patients

After the ED evaluation and treatment, 280 patients (68.6%) were discharged, and 128 (31.4%) were hospitalized. Hospitalization after ED consultation was voluntary in the vast majority of cases (*n* = 122, 97.6%), while involuntary admission was needed for 3 patients (2.4%). Hospitalization occurred at the two available psychiatric wards of the Città della Salute e della Scienza Hospital of the University of Turin, Italy.

Hospitalized and discharged patients did not differ in age (48.3 ± 17.7 years versus 45.2 ± 17.2 years, respectively; *t* = 1.65; *p* = 0.099). As shown in Table 5, those who were hospitalized were more frequently men, more frequently had a severe triage code, and more frequently reported a positive history of psychiatric illness, psychomotor agitation, delusions, euphoric mood, substance/alcohol intoxication, or suicidal ideation than those who were discharged. Nevertheless, it should be noted that 68 individuals evaluated as white/green during the triage code were hospitalized as well. Those who were discharged reported anxiety symptoms significantly more often than those who were hospitalized (see Table 5). No differences in LOS emerged between groups.

**Table 5:** Baseline differences between hospitalized versus non-hospitalized patients

	Total sample (*n* = 408)		
	Hospitalized patients *n* = 128	Discharged patients *n* = 280	Fisher’s exact test
	*n* (%)	*n* (%)	*p*
Gender			**0.024**
Women	60 (46.9)	166 (59.3)	
Men	68 (53.1)	114 (40.7)	
Nationality			0.928
Italian	115 (89.8)	252 (90)	
European Union	6 (4.7)	15 (5.4)	
Outside European Union	7 (5.5)	13 (4.6)	
Mode of ED arrival			0.125
Family members	46 (47.9)	75 (56.4)	
Ambulance personnel	34 (35.4)	49 (36.8)	
Law enforcement agency	7 (7.3)	4 (3)	
Others	9 (9.4)	5 (3.8)	
In charge of psychiatry service	76 (59.4)	144 (51.4)	0.164
Previous accesses to the ED	53 (41.4)	108 (38.6)	0.587
Triage code			** < 0.001**
Mild/White	2 (1.6)^a^	5 (1.8)^a^	
Moderate/Green	66 (51.6)^a^	207 (73.9)^b^	
Severe/Yellow	55 (43)^a^	65 (23.2)^b^	
Very severe/Red	5 (3.8)^a^	3 (1.1)^a^	
History of psychiatric illness	105 (82)	198 (70.7)	**0.015**
Length of stay, hours	7.2 (9.4)	6.2 (6.6)	0.261
Psychomotor agitation	24 (18.8)	18 (6.4)	** < 0.001**
Delusions	29 (22.7)	24 (8.6)	** < 0.001**
Hallucinations	8 (6.3)	7 (2.5)	0.086
Mood alterations	78 (60.9)	158 (56.4)	0.450
Mood polarity			**0.033**
Depressed mood	62 (48.4)^a^	134 (47.9)^a^	
Euphoric mood	7 (5.5)^a^	2 (0.7)^b^	
Dysphoria	9 (7)^a^	22 (7.9)^a^	
N/A	50 (39.1)^a^	122 (43.5)^a^	
Anxiety	67 (52.3)	214 (76.4)	** < 0.001**
Substance intoxication	20 (15.6)	16 (5.7)	**0.002**
Suicidal ideation	28 (21.9)	5 (1.8)	** < 0.001**

^a^Comparison of column proportions

^b^Comparison of column proportions

#### Associations between patients’ baseline features and psychiatric hospitalization

As shown in Table 6, several psychiatric conditions were significantly associated with emergency hospitalization, with suicidal ideation entailing the greatest risk. Across mood alterations, the euphoric mood was the condition most correlated with emergency hospitalization. In contrast, age, model of arrival, nationality, local catchment area, and time spent at the ED were not factors associated with hospitalization (see Table 6).

**Table 6:** Association between sociodemographic and clinical characteristics of the sample at ED admission and hospitalization

	Total sample (*n* = 408)		
	Wald’s test	*p*^a^	OR (95%CI)
Age, years	2.93	0.87	1.01 (0.99–1.02)
Male gender	5.43	**0.02**	1.65 (1.08–2.51)
Mode of ED arrival	4.90	0.179	
Nationality	0.14	0.931	
Local catchment area	0.06	0.801	
History of psychiatric illness	6.50	**0.011**	1.98 (1.2–3.3)
Psychomotor agitation	13.66	** < 0.001**	3.46 (1.8–6.7)
Delusions	13.71	** < 0.001**	3.06 (1.7–5.5)
Hallucinations	3.47	0.062	2.7 (0.9–7.7)
Presence of mood alterations (euphoric, dysphoric, depressed mood)	1.22	0.268	1.27 (0.83–1.97)
Euphoric versus dysphoric mood	5.84	**0.016**	8.9 (1.5–52.4)
Depressed versus dysphoric mood	0.518	0.472	1.37 (0.6–3.2)
Euphoric versus depressed mood	5.13	**0.024**	6.5 (1.3–32.8)
Anxiety	20.28	** < 0.001**	0.36 (0.2–.6)
Substance intoxication	11.28	**0.001**	3.35 (1.6–6.8)
Suicidal ideation	29.35	** < 0.001**	15.1 (5.6–40.3)
Hours spent at the ED	1.32	0.251	1.01 (0.98–1.04)

^a^Model corrected for gender

### Discharge as an outcome of the ED access

#### Clinical characteristics upon discharge from the ED

Approximately half of those receiving psychiatric consultation were administered pharmacotherapy (*n* = 217, 53.2%). More specifically, 128 individuals (59%) were given oral medications, 54 patients (24.9%) intravenous, and 10 (4.6%) intramuscular. Twenty-five patients (11.5%) received pharmacotherapy through multiple routes of administration. The vast majority of patients received anxiolytic medications (*n* = 170, 78.4%), 30 patients (13.8) received a combination of anxiolytics and antipsychotics, 13 individuals (6%) received antipsychotics, and 4 (1.8%) received a combination of > 2 classes of medications.

Out of the 283 patients who were discharged, the majority (*n* = 220, 78.6%) were sent to a psychiatrist for follow-up; the remaining patients were sent to their GPs (*n* = 47, 16.8%) and other specialists, including neurologists (*n* = 3, 1.1%) and other kinds of specialists (*n* = 10, 2.5%).

Out of those who were discharged from the ED, 328 (80.4%) were prescribed medications, in a substantial proportion of cases with a modified treatment plan: in 58% of patients taking anxiolytics, in 44.9% of patients using antidepressants, in 36.3% of those on antipsychotics, and in 18.9% of patients on mood-stabilizing medications.

## Discussion

In this investigation of LOS and the likelihood of hospitalization after ED admission for psychiatric conditions, two main findings emerged. First, the LOS was relatively short (mean of 6.5 h), highlighting an overall swift evaluation of patients with psychiatric symptomatology, and the main factor impacting a long LOS was substance/alcohol intoxication. Relatedly, when patients needed to be discharged, they also received an aftercare plan and, in a substantial number of cases, a revision of their pharmacological treatment. Second, upon ED arrival, hospitalized patients, when compared to those who were discharged, were more frequently men and more frequently reported a yellow/severe triage code, and more frequently had a positive history of psychiatric illness, psychotic symptoms, euphoric mood, or suicidal ideation. In line with these findings, the conditions most frequently leading to hospitalization were manic symptoms and suicidal ideation.

Given the current dearth of epidemiological data on psychiatric admissions to the ED [10], some findings are of note. Half of the patients were already in the care of a psychiatric service (53.9%). Coupled with the finding that patients with a positive psychiatric history represented the majority of the sample (74.3%), these results indicate that psychiatric consultation at the ED is probably not patients’ main first contact with psychiatrists, unlike other data worldwide have shown [10]. Our data are in contrast with data from other countries where gaps in community-based mental health services have been reported, where EDs consequently become providers of behavioral healthcare [23]. Relatedly, in another study, out of the 323,707 psychiatric patients being treated in their first contact in the past year, 93.4% started their treatment at the community-based mental health centers of the National Health System and not at the ED [1].

The leading cause of ED access was anxiety, followed by psychomotor agitation and depression. Since psychiatric presentations were not mutually exclusive (i.e., a patient could report anxiety and suicidal ideation), this is an expected finding, as anxiety accompanies a wide variety of psychiatric presentations. Nevertheless, anxiety was overrepresented when compared to the US data [12, 24, 25]. These findings could be partly explained by the proportion of patients with organic main symptoms at triage who then underwent a psychiatric evaluation.

Psychomotor agitation was also overrepresented (while psychosis was underrepresented) compared with earlier findings [12], possibly because this behavioral alteration may be an umbrella term for more specific diagnoses (i.e., psychosis, personality disorders). In contrast to data from Germany [26], alcohol-related disorders were less prevalent but were potentially associated with anxiety and psychomotor associations.

Psychoses were more poorly represented than in other studies [19]. A couple of hypotheses can be put forth to explain this finding. First, the community-based psychiatric service could lower patients' need for ED service, differently from what happens with patients reporting anxiety and depressive symptoms, who tend to be less frequently under the care of a psychiatric service. Second, given the setting of this study, one-fourth of the total sample was represented by patients reporting organic main symptoms at triage who then underwent a psychiatric evaluation, thus being more likely to show an anxious presentation. This tendency could be influenced by the fact that the Città della Salute e della Scienza hospital has a large catchment area, so it treats a high proportion of patients with organic conditions; in such a patient population, psychiatric consultations are more likely to arise from anxious and depressive symptoms than psychosis-like conditions.

The mean LOS was 6.5 h; after this time, only 28.5% of patients were still in the ED. Interestingly, this LOS is shorter than the ED LOSs in other countries; for example, data from the U.S. showed an LOS ranging from 6.7 to 11 h [14, 25], and those of Taiwan showed 17.6 ± 23.2 h [27]. However, data from European—and thus potentially more comparable—countries are sparse. Therefore, it would seem that, notwithstanding the great catchment area of the studied ED, the LOS can be deemed acceptable. Additionally, some specifiers emerged according to the patients’ main clinical presentation. For example, when considering the most life-threatening clinical presentations (i.e., suicidal ideation, psychomotor agitation, delusions), time to hospitalization was significantly shorter than it was for patients not reporting such symptomatology. Interestingly, the longest LOS was found in cases of substance/alcohol intoxication: a longer time is needed to perform all toxicological tests and related treatments, mostly delivered while patients are at the ED, without admission to the psychiatric ward [14]. In contrast, the shortest LOS (mean 7 h) was found for anxiety and suicidal ideation. Patients with anxiety had a significantly shorter LOS than those without anxiety. If we consider that anxiety is the leading cause of access to our ED, this could help explain the finding of a relatively shorter LOS than that in other countries. The second shortest LOS was that of patients with suicidal ideation requiring hospitalization, highlighting the need to keep these patients as safe as possible by providing them a specialized setting that lacks cords and sharp medical instruments.

Relatedly, LOS was independent of sociodemographic and clinical conditions. In fact, according to our findings, only alcohol/substance intoxication impacted LOS, in line with other studies [14, 26] and with real-world clinical practice given the effect on LOS of toxicology screening and related treatments. Additionally, given intoxicated patients’ frequent soporous condition, it may take a long time for the patient to be able to go through a psychiatric visit; sometimes a multidisciplinary (e.g., neurologist, anesthesiologist, internal medicine physician) and time-consuming approach is required. Importantly, our data provide support for studies reporting that LOS did not differ between hospitalized and discharged patients, although this is a debated topic [14, 15].

Our findings confirm the importance of patients’ clinical presentation when a decision about hospitalization has to be made. It is noteworthy that 31% of patients were hospitalized after psychiatric consultation at the ED, which is in line with the Italian data (25.8%) but different from those reported in the U.S. (up to 58% [2]). We found suicidal ideation as the main clinical factor associated with being hospitalized after seeking help at the ED (OR = 15.1), followed by euphoric mood and psychotic symptoms (i.e., hallucinations and delusions), in line with previous data [24]. Interestingly, only clinical data and not predisposing (i.e., age) or system-related factors (i.e., arrival by emergency transport) reached significance in this model, unlike earlier findings [24]. Taken together, these findings are of clinical interest since specific paths could be implemented for those seeking help at the ED with psychotic, euphoric or suicidal psychiatric presentation.

When considering these findings, some strengths should be considered. First and foremost, no insurance/monetary barriers were present, thus providing much needed [2, 10] real-world data with an epidemiological focus and a large sample size. Our study suffers from some limitations as well. The retrospective design of this study hampers our ability to make causal inferences. The overall LOS was considered, without specification of subsections of this time (i.e., time from door to request, time from request to the psychiatric evaluation, time from the start of the psychiatric evaluation to the disposition decision, time from the disposition decision to discharge). This is a one-site study, so the data generalizability might be lacking. Organic pathologies such as encephalitis, metabolic or endocrine disorders were not recorded, nor was the type of substance causing intoxication. That said, given the vastness of this topic and its impact on patients and clinicians alike, further studies are needed to improve the quality of the data on psychiatric patients at the ED and provide standardized assessments (e.g., SADPERSON scale).

In closing, our findings suggest that anxiety, psychomotor agitation, and depression were the most frequent causes of ED presentation; LOS was overall short and was mainly influenced by alcohol/substance intoxication. Finally, hospitalization was associated mostly with clinical conditions upon admission, particularly suicidal ideation, and not predisposing (e.g., age) or system-related factors (e.g., mode of arrival). It is noteworthy that psychiatric patients were evaluated in a reasonable amount of time and that, even for those who were discharged, in the vast majority of cases, follow-up assessments were planned and medication schemes were revised, meaning their treatment entailed not a mere resolution of the emergency condition.
